# Asexual Experimental Evolution of Yeast Does Not Curtail Transposable Elements

**DOI:** 10.1093/molbev/msab073

**Published:** 2021-03-15

**Authors:** Piaopiao Chen, Jianzhi Zhang

**Affiliations:** Department of Ecology and Evolutionary Biology, University of Michigan, Ann Arbor, MI, USA

**Keywords:** transposon, excision, asexual reproduction, sexual reproduction, mutation accumulation, *Saccharomyces cerevisiae*

## Abstract

Compared with asexual reproduction, sex facilitates the transmission of transposable elements (TEs) from one genome to another, but boosts the efficacy of selection against deleterious TEs. Thus, theoretically, it is unclear whether sex has a positive net effect on TE’s proliferation. An empirical study concluded that sex is at the root of TE’s evolutionary success because the yeast TE load was found to decrease rapidly in approximately 1,000 generations of asexual but not sexual experimental evolution. However, this finding contradicts the maintenance of TEs in natural yeast populations where sexual reproduction occurs extremely infrequently. Here, we show that the purported TE load reduction during asexual experimental evolution is likely an artifact of low genomic sequencing coverages. We observe stable TE loads in both sexual and asexual experimental evolution from multiple yeast data sets with sufficient coverages. To understand the evolutionary dynamics of yeast TEs, we turn to asexual mutation accumulation lines that have been under virtually no selection. We find that both TE transposition and excision rates per generation, but not their difference, tend to be higher in environments where yeast grows more slowly. However, the transposition rate is not significantly higher than the excision rate and the variance of the TE number among natural strains is close to its neutral expectation, suggesting that selection against TEs is at best weak in yeast. We conclude that the yeast TE load is maintained largely by a transposition–excision balance and that the influence of sex remains unclear.

## Introduction

Transposable elements (TEs) are mobile DNA fragments that are abundant in the genomes of nearly all eukaryotes (Wicker et al. 2007). Their success is attributable to their ability to self-replicate independently of their host genomes. Although TE transpositions occasionally lead to adaptations (Schrader and Schmitz 2019), they more often disrupt functional genes and promote chromosomal rearrangement via ectopic recombination (Kidwell and Lisch 2001; Bartolomé et al. 2002; Rizzon et al. 2002; Petrov et al. 2003). Therefore, TEs are believed to be selectively purged as a result of their negative fitness impact on the host. Certain types of TE can be excised through homologous recombination between its flanking long terminal repeats (LTRs), resulting in a solo LTR left in the genome (Farabaugh and Fink 1980; Jordan and McDonald 1999).

Sex has long been hypothesized to impact TE’s evolutionary success. On the one hand, sexual reproduction allows TEs to colonize new genomes during zygote formation, facilitating their spread from one individual to the entire population (Hickey 1982; Zeyl et al. 1996). Because the more active a TE is, the higher the chance that it will colonize a new genome, sex favors the emergence of highly active TEs (Charlesworth and Langley 1986). In the same vein, in asexual lineages, TEs are expected to become less active and may even go extinct given their negative fitness impact on the host (Wright and Finnegan 2001). On the other hand, chromosome assortment and recombination during meiosis enhances the efficacy of selective purging of deleterious TEs, limiting their proliferation. By contrast, TEs are expected to accrue in asexual lineages through Muller’s ratchet (Muller 1964). Thus, it is theoretically unclear whether the net impact of sex on TE’s success is positive or negative. A further complication is the influence of the effective population size (*N*_e_), because Muller’s ratchet runs faster and selection for mechanisms containing TEs becomes weaker under a smaller *N*_e_; consequently, an asexual lineage with a small *N*_e_ may go extinct because of TEs, whereas that with a large *N*_e_ may be able to evolve mechanisms to contain TEs (Dolgin and Charlesworth 2006). Empirically, several studies have attempted to address this question by comparing the TE load in natural sexual and related asexual lineages. For example, bdelloid rotifers, which have abandoned sexual reproduction millions of years ago (Welch and Meselson 2000), are known to have lost all active retrotransposons (Arkhipova and Meselson 2000, but see Nowell et al. 2021). However, there are also instances where sexual and related asexual lineages show no significant difference in TE prevalence (Ågren et al. 2015; Bast et al. 2016). Furthermore, a comparison of the TE load between outcrossing and selfing plant species revealed no clear difference (Slotte et al. 2013). A broad phylogenetic survey of animal genomes was inconclusive regarding the impact of sex on the genomic TE load (Jaron et al. 2021). Note that because (asexual) lineages that have gone extinct owing to unbearable TE loads could not be studied, comparative studies are subject to a potential ascertainment bias.

Given the ambiguous results from comparative studies, Bast et al. (2019) recently turned to genomic data generated from experimental evolution of the yeast *Saccharomyces cerevisiae* (McDonald et al. 2016), because yeast can reproduce both sexually and asexually depending on the environmental cue. More importantly, controlled experiments have advantages over comparative studies in several aspects such as the lack of the mentioned ascertainment bias and the certainty in the environment, reproductive mode, evolutionary time, and ancestral genome. The yeast genome has five families of TEs, Ty1 through Ty5, all being retrotransposons flanked by LTRs (Kim et al. 1998; Carr et al. 2012). These TEs, comprising over 400 solo LTRs resulting from excisions and about 50 full-length TEs, collectively constitute approximately 3.1% of the yeast genome (Carr et al. 2012). Bast et al. (2019) reported that the yeast genomic TE load remained constant in sexual populations but rapidly decreased in asexual populations over about 1,000 generations of experimental evolution, therefore concluding that sex is required for the evolutionary maintenance of TEs in yeast. They proposed and supported by population genetic simulation a hypothesis that the observed TE load reduction during asexual experimental evolution was caused by the rise of modifiers that increase the TE excision rate. Because the benefit of such a modifier is entirely in its reduction of the genomic TE load, it has a greater selective advantage in asexual organisms where it is linked with the lowered TE load, than in sexual organisms where its association with the lowered TE load is broken by recombination. Consequently, the modifiers are more likely to be fixed and have greater impacts on the TE load in asexual populations. Notwithstanding, Bast et al.’s results raise doubts for four reasons. First, their observation that numbers of full-length TEs and solo LTRs both decreased by ∼30% after ∼1,000 asexual generations cannot be fully explained by increased excisions, because solo LTRs cannot be excised except for those neighboring solo LTRs with virtually no intervening functional sequence. Second, their simulation assumed that the initial frequency of the modifier was at least 1%, which seems unrealistic given the effective population size of 10^5^ in the experimental evolution that started from a single clone (McDonald et al. 2016). Third, in natural populations (as opposed to lab strains), yeast undergoes approximately one sexual cycle per 1,000 asexual generations (Ruderfer et al. 2006; Tsai et al. 2008). If Bast et al.’s results were correct, natural yeast must recover in one sexual cycle the 30% TE loss from the preceding 1,000 asexual generations to maintain the observed TE load; it is difficult to imagine how this could be accomplished. Fourth, it is important to recognize that even the performed sexual experimental evolution was largely asexual, because yeast was forced to undergo only one sexual cycle every 90 asexual generations (McDonald et al. 2016). In other words, both the sexual and asexual lineages in the experimental evolution had similar life cycles as yeast’s natural life cycle, with the asexual lineage resembling nature more closely. It is thus puzzling why the yeast TE load appeared to deviate from its natural load in asexual but not sexual experimental evolution.

In this work, we investigate TE dynamics under sexual and asexual reproduction by analyzing genomic data from multiple yeast experimental evolution studies, including the one considered by Bast and colleagues. We further estimate TE transposition and excision rates in the near absence of natural selection using yeast mutation accumulation (MA) lines. Our analyses suggest that 1) Bast et al.’s result is unreliable due to low genomic sequencing coverages, 2) asexual experimental evolution does not lower yeast’s TE load, 3) this load is maintained largely by a transposition–excision balance with minimal influences of selective purging of TEs, and 4) the net impact of sex on TE’s evolutionary success remains unclear.

## Results

### Bast et al.’s Genomic Observations Appear Unreliable Due to Low Sequencing Coverages

**Fig. 1.**
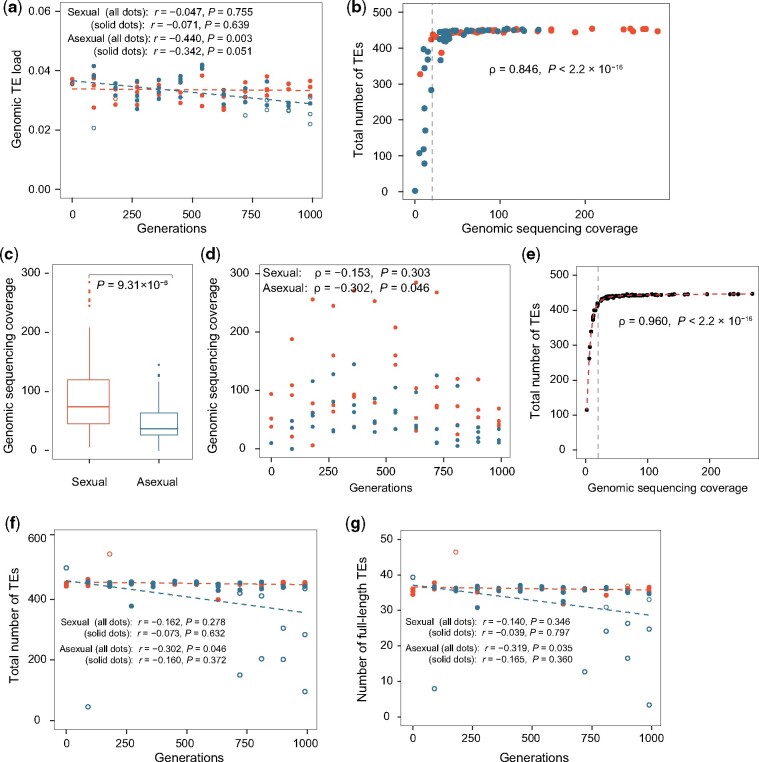
Reanalysis of McDonald et al.’s data previously analyzed by Bast et al. shows that asexual experimental evolution does not curtail yeast TEs upon the removal of unreliable samples. Unless otherwise noted, red and blue indicate sexual and asexual populations, respectively. (*a*) The genomic TE load at various time points in sexual and asexual experimental evolution. Solid and open dots show samples with >20× and <20× genomic sequencing coverage, respectively. Pearson’s correlation (*r*) between TE load and number of generations of evolution is shown along with the *P* value. Dashed lines show linear regressions. (*b*) Relationship between the observed TE number and the genomic sequencing coverage across samples. The vertical line indicates the coverage of 20. Spearman’s rank correlation (*ρ*) between the coverage and TE number is shown along with the *P* value. (*c*) Genomic sequencing coverage is significantly higher for sexual populations than asexual populations. In each box plot, the lower and upper edges of a box represent the first (qu_1_) and third (qu_3_) quartiles, respectively, the horizontal line inside the box indicates the median (md), the whiskers extend to the most extreme values inside inner fences, md±1.5(qu_3_−qu_1_), and the circles represent values outside the inner fences (outliers). The *P* value is based on Wilcoxon rank-sum test. (*d*) Genomic sequencing coverages of individual samples. The *ρ* between coverage and number of generations of evolution is shown along with the *P* value. (*e*) Numbers of TEs detected from individual subsamples created by downsampling the reads from sexual population 2F5F at 630 generations to the coverage levels of all real samples. The red dashed line is the SCAM regression. The vertical line indicates the coverage of 20. The *ρ* between the coverage and TE number is shown along with the *P* value. (*f* and *g*) Estimated numbers of TEs (*f*) and full-length TEs (*g*) of individual samples upon the regression-based correction for low coverage. All symbols follow panel (*a*).

The data (supplementary table S1, Supplementary Material online) analyzed by Bast et al. (2019) were generated by McDonald et al. (2016), who evolved four sexual and four asexual yeast lines for 990 generations. Meiosis was induced in sexual lines after every 90 asexual generations. Whole populations were sequenced at every 90 generations for all lines. Following Bast et al. (2019), we used two approaches to probe TE load changes during the experimental evolution. In the first approach, we quantified from each sample the genomic TE load, which is the fraction of the genome occupied by TEs. Operationally, we estimated the genomic TE load by the weighted number of bases stemming from TE regions divided by the weighted number of bases of the genome, where the weight of a base is the number of hits it receives from Illumina sequencing reads. Our estimator is expected to be slightly more accurate than the fraction of reads mapped to TEs, which Bast et al. (2019) used to estimate the TE load, because a read could span the boundary between a TE region and its neighboring non-TE region. Note that different types of TEs such as full-length TEs and solo LTRs are not differentiated in this approach. Because the yeast mitochondrial genome does not harbor any TE but the mitochondrial DNA copy number may vary among samples, we discarded all reads mapped to mitochondrial DNA. Similar to what was discovered by Bast et al. (2019), we found the genomic TE load to be stable in sexual populations but significantly decreased over time in asexual populations (fig. 1*a*). When the four asexual populations were separately examined, the TE load decreased over time in all four populations, although this trend was significant in only two of them (supplementary fig. S1, Supplementary Material online).

Because the above approach did not separate individual TE insertions, we adopted the second approach, which detects the presence/absence of specific TEs. We initially used the tool McClintock (Nelson et al. 2017), which Bast et al. (2019) employed, but found a number of false-positive and false-negative errors (see Materials and Methods). After some comparisons, we decided to use RelocaTE2, which identifies TE transposition sites from next-generation sequencing data with demonstrated sensitivity and specificity (Chen et al. 2017); an earlier version of this program was part of McClintock (Nelson et al. 2017). Because TE detection is influenced by the genomic sequencing coverage (Bast et al. 2019), we examined the coverages of individual samples (supplementary table S2, Supplementary Material online). We observed a sharp rise in the number of TEs detected as the genomic sequencing coverage increases from 0 to 20; the TE number plateaus when the coverage exceeds 20 (fig. 1*b*). In addition, we found that the asexual lines had significantly lower coverages than the sexual lines (fig. 1*c*) and that the coverage decreased significantly with the number of generations of evolution for asexual but not sexual populations (fig. 1*d*). Hence, it is possible that the observed decline of the TE load in asexual experimental evolution is an artifact. To investigate quantitatively the effect of sequencing coverage on TE detection, we 1) chose the sample with the highest genomic sequencing coverage (2F5F at 630 generations), 2) respectively randomly downsampled the raw reads in this sample to the coverages of all other samples, and 3) identified TEs from these randomly created subsamples. We performed ten downsamplings from the same original sample and found the number of detected TEs to be highly similar for samples with the same coverage. As expected, the number of detected TEs from a subsample increased with its coverage (fig. 1*e*). Importantly, the TE count was drastically below the true value when the coverage was below 20 (fig. 1*e*). Similar trends were observed for full-length TEs, reference TEs (TEs present in the reference genome), and nonreference TEs (TEs absent from the reference genome) (supplementary fig. S2*a*–*c*, Supplementary Material online). Using a fitted regression between the number of TEs detected from a subsample and the coverage of the subsample (fig. 1*e*), we calculated a correction factor for each coverage level by dividing the number of TEs detected from the original sample by the number of TEs detected in the subsample with that coverage. Correction factors were similarly computed for full-length TEs, reference TEs, and nonreference TEs from their respective regression curves (supplementary fig. S2*a*–*c*, Supplementary Material online).

We then estimated the number of TEs from an actual sample by multiplying the number of TEs detected from the sample by the correction factor corresponding to the coverage of the sample. We found that the estimated TE number remained constant over time in sexual lines, but decreased in asexual lines (fig. 1*f*). In three of the four asexual lines, the decrease was significant (supplementary fig. S2*d*, Supplementary Material online). Similar results were obtained when only full-length TEs were considered (fig. 1*g* and supplementary fig. S2*e*, Supplementary Material online). Notwithstanding, we noticed that the estimated numbers of TEs and full-length TEs from samples with very low (<20) coverages tend to be outliers (fig. 1*f* and *g*; supplementary fig. S2*d* and *e*, Supplementary Material online) when compared with those from other samples, suggesting that the corrections made in TE number estimation were insufficient or ineffective. This is possible, because very low coverages could be caused by low sequencing depths, DNA degradation, poor library preparation, and other reasons, but our correction assumed that the coverage is entirely determined by the sequencing depth. Consistent with this possibility is the observation that the TE number drops more precipitously in figure 1*b* than in figure 1*e* as the genomic sequencing coverage reduces from 20. To further investigate this possibility, we examined the relationships between the genomic sequencing coverage and three measures of data quality: mean fragment size of the sequencing library, faction of mapped reads, and mean base quality (supplementary fig. S3*a*–*c*, Supplementary Material online). In all three cases, we observed lower data qualities for samples with lower sequencing coverages, and almost every sample with the corrected TE number in the bottom 20% of all samples shows relatively low data qualities. Furthermore, we found that a small fragment size very negatively impacts the estimate of the corrected TE number (supplementary fig. S3*d*, Supplementary Material online). Given these findings and the lack of properly corrected estimates of TE numbers for samples with <20× coverage, we excluded them from the analysis. Consequently, in neither sexual nor asexual experimental evolution did we find the TE number to change significantly (fig. 1*f*), and this result was further confirmed in individual asexual populations (supplementary fig. S2*d*, Supplementary Material online). The same was true for full-length TEs (fig. 1*g* and supplementary fig. S2*e*, Supplementary Material online). We similarly removed the unreliable samples from the analysis of the genomic TE load, because a low coverage caused by factors other than a low sequencing depth could also affect TE load estimation. We confirmed that now neither sexual nor asexual experimental evolution showed a significant change in TE load over time (fig. 1*a* and supplementary fig. S1, Supplementary Material online). Note that, although Bast et al. (2019) corrected their estimates of TE numbers according to the sequencing coverage similar to what we did, they did not exclude the unreliable samples from their analysis. Taken together, our analyses suggest that the previously reported decrease of yeast TE load and TE number in asexual experimental evolution was likely an artifact of inaccurate TE estimation from genomic data of very low coverages.

### Bast et al.’s Simulation Is Untenable

**Fig. 2.**
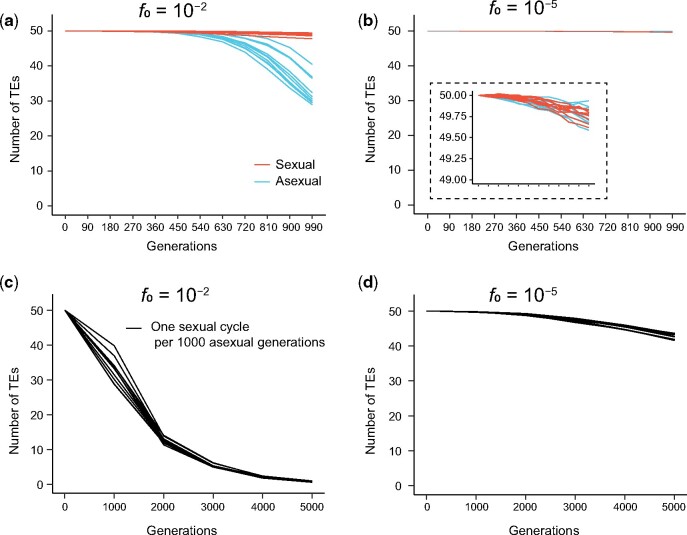
Population genetic simulation of Bast et al.’s model under various parameter settings. (*a*) Under the initial allele frequency (*f*_0_) of 10^−2^ for the modifier that increases the excision rate, TE numbers reduce more in asexual (blue) than sexual (red) populations. Each line shows one simulation replication, and ten replications were simulated for sexual and asexual populations, respectively. A sexual population undergoes one sexual cycle after every 90 asexual generations. (*b*) Under the more realistic *f*_0_ of 10^−5^, TE numbers change only slightly in 1,000 generations, and no significant difference is detected between sexual and asexual populations (see inset). A sexual population undergoes one sexual cycle after every 90 asexual generations. (*c* and *d*) When sexual reproduction occurs once after 1,000 asexual generations as in natural yeast populations, TE numbers continuously drop under *f*_0_=10^−2^ (*c*) or 10^−5^ (*d*), indicating that the model is unrealistic.

As mentioned, Bast et al. (2019) performed a population genetic simulation to demonstrate that their observed decrease of the TE number in asexual experimental evolution is explainable by the rise of an excision rate modifier. The authors assumed that the modifier allele had an initial frequency (*f*_0_) of at least 1%, which is unrealistically high given the effective population size of 10^5^ in the simulation (McDonald et al. 2016) as well as the experimental evolution that started from a single clone (McDonald et al. 2016). It is thus relevant to investigate whether their simulation result is robust to *f*_0_. We first repeated their simulation under *f*_0_* *=* *0.01, and observed essentially no TE number change over 1,000 generations in sexual populations (where sex occurred once every 90 asexual generations) but a reduction of the TE number by 20–30% in asexual populations (fig. 2*a*), similar to what Bast et al. (2019) reported. We then lowered *f*_0_ to 10^−5^, representing newly arising mutations in the simulated populations as well as experimental evolution populations. Now, in both sexual and asexual populations, TE numbers hardly changed over 1,000 generations, and the difference between the ten sexual and ten asexual populations was not significant (*P *=* *0.791, Wilcoxon rank-sum test) (fig. 2*b*). Thus, Bast et al.’s model cannot explain the purported TE load disparity between sexual and asexual experimental evolution.

We also assessed Bast et al.’s model under the scenario of one sexual cycle after every 1,000 asexual generations, as in natural populations of yeast. Regardless of whether *f*_0_* *=* *0.01 or 10^−5^, we found that one sexual cycle cannot recover the TEs lost from 1,000 asexual generations. In merely 5,000 generations, which is on the order of a few years for yeast, all TEs were lost if *f*_0_* *=* *0.01 (fig. 2*c*). Under the more realistic *f*_0_ of 10^−5^, nearly 20% of TEs were lost in 5,000 generations (fig. 2*d*). Clearly, these results demonstrate that Bast et al.’s model cannot explain the observed TE number in natural populations of yeast. Given the untenability of Bast et al.’s model, the simulation results cannot be used to support or explain the findings from the yeast experimental evolution.

### Stable TE Numbers in Additional Sexual and Asexual Experimental Evolution Studies

**Fig. 3.**
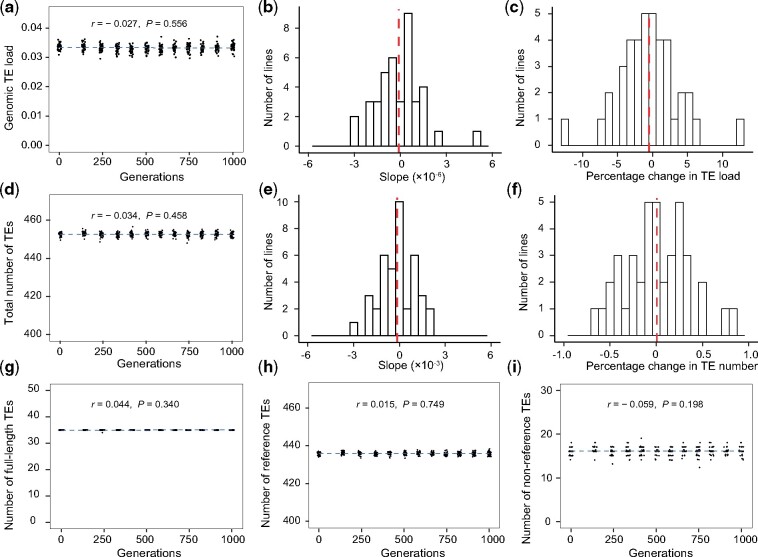
Yeast genomic TE load and numbers of various groups of TEs are stable over Lang et al.’s asexual experimental evolution. (*a*) Genomic TE load remains stable in 40 asexual lines over 1,000 generations. Each dot represents a line at a time point. The linear regression of TE load over 12 time points for all data points is shown, along with Pearson’s correlation (*r*) and *P* value. (*b*) Frequency distribution of the slope of the 40 linear regressions of genomic TE load on number of generations, one per experimental evolution line. The red dashed vertical line indicates the mean of the distribution. (*c*) Frequency distribution of the percentage change in TE load per line from the start to the end of experimental evolution. The red dashed vertical line indicates the mean of the distribution. (*d*) Total numbers of TEs remain stable in 40 asexual lines over 1,000 generations. Symbols follow panel (*a*). (*e*) Frequency distribution of the slope of the 40 linear regressions of number of TEs on number of generations, one per experimental evolution line. The red dashed vertical line indicates the mean of the distribution. (*f*) Frequency distribution of the percentage change in TE number per line from the start to the end of experimental evolution. The red dashed vertical line indicates the mean of the distribution. (*g*–*i*) Numbers of full-length TEs (*g*), reference TEs (*h*), and nonreference TEs (*i*) remain stable in 40 asexual lines over 1,000 generations. Symbols follow panel (*a*).

As described, no significant changes in TE load or TE numbers were found in the experimental evolution data analyzed by Bast et al. upon the removal of unreliable samples. Because this result could be due to an insufficient statistical power, we analyzed the genomic data from two additional yeast experimental evolution studies (Lang et al. 2013; Leu et al. 2020). In the first study, Lang et al. (2013) evolved 40 asexual yeast populations in rich medium for 1,000 generations and sequenced each population to 100× coverage at 12 time points (supplementary table S1 and fig. S4*a*, Supplementary Material online). We first quantified the genomic TE load after removing all reads mapped to mitochondrial DNA. No significant correlation was found between the TE load and the number of generations of evolution when the 40 lines were analyzed together (fig. 3*a*). When the 40 lines were individually examined, exactly one half showed a negative correlation (regardless of statistical significance) (fig. 3*b*). The mean TE load of the 40 lines was 0.0337 ± 0.0003 (mean ± 95% confidence interval) at the start and 0.0335 ± 0.0004 at the end of the experimental evolution. The fraction of lines with a decreased TE load (60%) based on the start and end of evolution did not deviate significantly from the random expectation (*P* = 0.268, two-tailed binomial test). The percentage change in TE load averaged across the 40 lines was not significantly different from 0 (*P *=* *0.479, *t*-test) (fig. 3*c*).

Next, we identified individual TEs using RelocaTE2. We first performed downsampling from the sample with the highest sequencing coverage (BYS1_E03 at 1,000 generations) to investigate the impact of coverage on the number of detected TEs in this data set. Numbers of detected TEs, reference TEs, and nonreference TEs all rose with the coverage (supplementary fig. S4*b*–*d*, Supplementary Material online), whereas the number of detected full-length TEs did not change with the coverage (supplementary fig. S4*e*, Supplementary Material online). The standard deviation of the number of detected TEs among the subsamples from downsampling was quite small here (1.04), compared with the corresponding value for McDonald et al.’s data (44.6). Furthermore, the sequencing coverage was not significantly correlated with the number of generations of evolution (supplementary fig. S4*a*, Supplementary Material online). Hence, we expect the results from this data set to be more reliable than Bast et al.’s results. After correcting the effect of sequencing coverage, we found no significant correlation between the number of TEs and the number of generations of evolution (fig. 3*d*). The fraction (52.5%) of lines exhibiting a negative correlation (regardless of statistical significance) between the TE number and number of generations of evolution was not significantly different from the chance expectation of 50% (*P *=* *0.875, two-tail binomial test) (fig. 3*e*). We detected on average 452.48 ± 0.31 and 452.51 ± 0.37 TEs at the start and end of the experimental evolution, respectively. By comparing the start with the end of the evolution for each line, we observed exactly one half of the 40 lines to decrease in TE number. The percentage change in TE number averaged across the 40 lines was not significantly different from 0 (*P *=* *0.917, *t*-test) (fig. 3*f*). Furthermore, the numbers of full-length TEs, reference TEs, and nonreference TEs did not correlate significantly with the number of generations of evolution (fig. 3*g*–*i*).

**Fig. 4.**
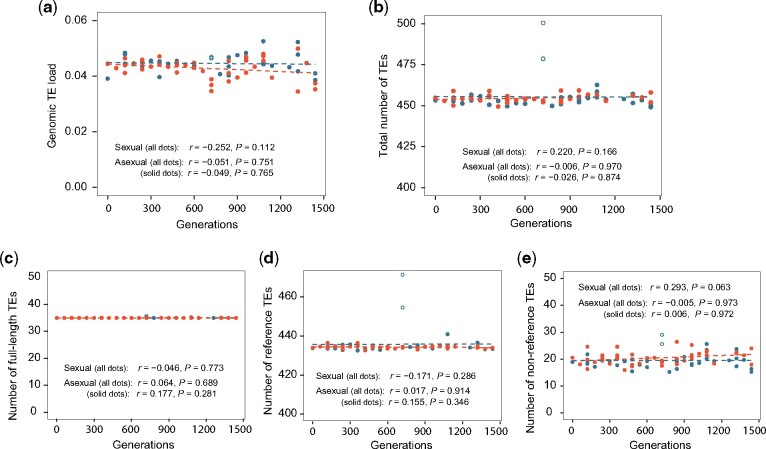
Yeast genomic TE load and numbers of various groups of TEs are stable over Leu et al.’s sexual and asexual experimental evolution. Red and blue dots indicate sexual and asexual populations, respectively, whereas solid and open dots show samples with >20× and <20× genomic sequencing coverage, respectively. (*a*) Genomic TE load remains stable in three sexual and three asexual lineages over 1,440 generations. The linear regression of TE load over the number of generations of experimental evolution is shown, along with Pearson’s correlation (*r*) and *P* value. (*b*–*e*) Numbers of all TEs (*b*), full-length TEs (*c*), reference TEs (*d*), and nonreference TEs (*e*) of individual samples upon the regression-based correction for low coverage. Symbols follow panel (*a*).

In the second study, Leu et al. (2020) evolved six sexual (one sexual cycle per 60 asexual generations) and six asexual lines of yeast originating from a diploid ancestor (W303) in a fluctuating environment for 1,440 generations. Six of the 12 lines (3 sexual* *+* *3 asexual) were sequenced to an average of 80× coverage at multiple time points (supplementary table S1, Supplementary Material online). We quantified the genomic TE load after removing all reads mapped to mitochondrial DNA. In neither sexual nor asexual experimental evolution did we observe a significant correlation between the TE load and the number of generations of evolution (fig. 4*a*). When examining individual lines, we found no significant correlation for any sexual line, but found one asexual line to show a significantly negative correlation (nominal *P *=* *0.035) (supplementary fig. S5*a*, Supplementary Material online). But this significance disappeared after the correction for multiple testing. In Leu et al.’s data, a few samples were sequenced to <20× coverage, and sexual populations tended to have higher coverages than asexual populations, although their difference was not statistically significant (supplementary fig. S5*b*, Supplementary Material online). After we removed samples with <20× coverage, the results in figure 4*a* and supplementary figure S5*a*, Supplementary Material online, remained qualitatively unchanged.

When identifying individual TEs from Leu et al.’s data using RelocaTE2, we similarly performed the downsampling analysis from the sample with the highest coverage (sexual_1 at 1,440 generations). The effects of sequencing coverage on the number of detected TEs, full-length TEs, reference TEs, and nonreference TEs were all significant (supplementary fig. S5*c*–*f*, Supplementary Material online). The standard deviation in TE number among subsamples resulting from downsampling is 7.43, suggesting that the reliability of the results from the present data probably sits between the reliabilities of the results from McDonald et al.’s data and from Lang et al.’s data. After correcting the effect of sequencing coverage, we found that the total number of TEs is not significantly correlated with the number of generations of evolution in sexual or asexual lines (fig. 4*b*). We also observed no significant correlation in each line either including or excluding samples with low sequencing coverages (<20) (supplementary fig. S5*g*, Supplementary Material online). Furthermore, numbers of full-length TEs, reference TEs, and nonreference TEs were not significantly correlated with the number of generations of evolution in sexual or asexual lines (fig. 4*c*–*e*).

Together, the analyses of data from these two additional experimental evolution studies confirm the results from our reanalysis of McDonald et al.’s data that the TE load in the yeast genome is stable in sexual as well as asexual lines.

### Rates of TE Transposition and Excision during Asexual MA

Because the evolution of the genomic TE load is potentially impacted by both mutation and selection, knowing the rate of change of the TE load in the absence of selection is critical for understanding its evolution. Below, we estimate TE transposition and excision rates from a recently published yeast MA study where random mutations were accumulated in seven different media (24 diploid lines per medium) for 1,000 asexual generations in the near absence of selection (Liu and Zhang 2019). We used the published sequencing data (supplementary table S1, Supplementary Material online) at both the start and end of MA to quantify TE numbers by RelocaTE2. The paired-end DNA libraries had an average insert size of about 400 bases and were sequenced to a depth of 80× genomic coverage (Liu and Zhang 2019). In total, we observed 21 TE transpositions from the 167 MA lines (one of the 168 lines died out during MA) (supplementary table S3, Supplementary Material online). We confirmed the transpositions by loading the mapped reads on Integrated Genomics Viewer (IGV) (supplementary fig. S6*a*, Supplementary Material online). We also detected 41 deletions, but found that they were all localized to the same locus and all of the deleted fragments have 2,404 bases including genes *MET15* and *YLR302C* (supplementary fig. S6*b*, Supplementary Material online). Because the progenitor strain (BY4743) used in the MA is heterozygous for *MET15* deletion (*met15Δ0*/*MET15*), we hypothesize that the parallel deletions of this fragment in 41 lines resulted from loss of heterozygosity (LOH). Under this hypothesis, we should also observe the homozygous presence of *MET15* (*MET15*/*MET15*) in some lines. Indeed, 36 lines showed the homozygosity of *MET15* (supplementary fig. S6*b*, Supplementary Material online). Furthermore, the proportions of lines with only the *MET15* allele and lines with only the *met15Δ0* allele are not significantly different (*P *=* *0.649, two-tail binomial test). Together, these observations suggest that the 41 deletions were not true excisions.

In the above analysis, we realized that because the MA genomic data were from diploids, we must distinguish the homozygous from heterozygous presence of a TE in order to identify TE losses. Unfortunately, this task cannot be accomplished by most tools for TE detection, including RelocaTE2. We thus developed our own method that uses the information of the two reads in a read pair to identify TE losses (supplementary fig. S6*c*, Supplementary Material online) (see Materials and Methods). We verified the accuracy of our method using known heterozygous (*MET15* and *LYS2*) and homozygous (*URA3*, *LEU2*) gene deletions in BY4743. Our method identified 15 genuine TE excisions in the 167 MA lines (supplementary table S3, Supplementary Material online). Note that the genomic data of MacDonald et al. and those of Lang et al. were from haploids. Although the genomic data of Leu et al. were from diploids, applying the above method did not alter the results in figure 4.

**Fig. 5.**
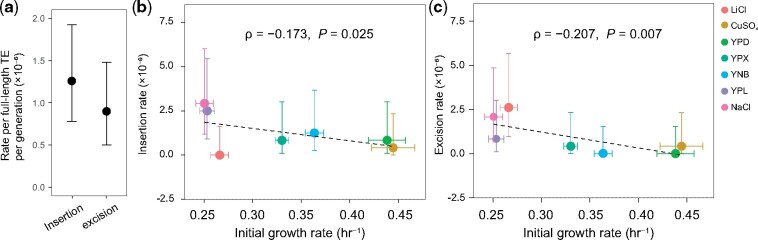
TE transpositions and excisions in 167 yeast mutation accumulation (MA) lines collected in seven different environments. (*a*) Estimated rates of TE transposition and excision per full-length TE per generation. The error bar shows the 95% confidence interval. The transposition and excision rates are not significantly different (*P *=* *0.36, Wilcoxon rank-sum test). (*b* and *c*) Both rates of TE transposition (*b*) and excision (*c*) per full-length TE per generation in an environment decrease with the yeast growth rate in the environment before the MA. The black dashed line shows the linear regression. Spearman’s rank correlation and *P* value are shown. Vertical and horizontal error bars respectively show standard deviations of transposition/excision rates and growth rates among replicate lines.

Based on the 21 transposition events in the MA data, we estimated that the rate of TE transposition is *u *=* *1.26 × 10^−6^ (95% confidence interval: 7.78 × 10^−7^–1.92 × 10^−6^) per full-length TE per generation (fig. 5*a*; see Materials and Methods), which is two orders of magnitude lower than that estimated in *Drosophila* (Maside et al. 2000; Adrion et al. 2017). The transposition rate ranges from 0 (in LiCl) to 5.833 × 10^−6^ (in NaCl) among the seven environments. Interestingly, we observed a significant, negative correlation between the TE transposition rate in an environment and the yeast growth rate (before MA) in the environment (Spearman’s ρ* *=* *−0.173, *P *=* *0.025) (fig. 5*b*). The trend of higher TE transposition rates in environments of lower yeast fitness is similar to what was previously observed for point mutations and small insertions/deletions (Liu and Zhang 2019).

Based on the 15 excisions in the MA data, we estimated that the rate of TE excision is *v *=* *8.98 × 10^−7^ (95% confidence interval: 5.03 × 10^−7^–1.48 × 10^−6^) per full-length TE per generation (fig. 5*a*), about one order of magnitude lower than that estimated in *Drosophila* (Maside et al. 2000; Adrion et al. 2017). Interestingly, the excision rate in an environment is also significantly negatively correlated with the yeast growth rate in the environment (ρ* *=* *−0.207, *P *=* *0.007) (fig. 5*c*). However, *u–v* is not significantly correlated with the growth rate (ρ = 0.001, *P *=* *0.99), allowing us to infer the neutral rate of TE number changes without considering the specific environment.

### Testing Selection against Yeast TEs

It is reasonable to assume that the present-day TE load in yeast is at an equilibrium; this assumption is consistent with the lack of significant changes in TE load in experimental evolution. Because our estimates of *u* and *v* are not significantly different (*P *>* *0.3, Wilcoxon rank-sum test) (fig. 5*a*), it is possible that the yeast TE load is maintained simply by a balance between transpositions and excisions without the involvement of selection against TEs. To further test this idea, we compared the variance in TE number among natural strains of yeast (*V*_g_) with that generated by mutation per generation (*V*_m_). Under neutrality, *V*_g_/*V*_m_ is expected to equal 4 *N*_e_ for primarily asexual diploids like yeast (Lynch and Hill 1986; Denver et al. 2005). Purifying selection against TEs would render *V*_g_/*V*_m_ smaller than the neutral expectation. We estimated *V*_g_ based on all TEs previously identified from 41 natural *S. cerevisiae* strains of diverse ecological and geographic origins (Bleykasten-Grosshans et al. 2013) and estimated *V*_m_ from the 167 MA lines. We found *V*_g_/*V*_m_ to equal 1.51 × 10^7^, which is slightly below the neutral expectation of 4 *N*_e_* *=* *4 × 10^7^ because yeast’s *N*_e_ is about 10^7^ (see Materials and Methods). This result is consistent with the notion that selection against yeast TEs is minimal. The inverse of *V*_g_/*V*_m_, or 6.6 × 10^−8^, equals the coefficient of selection (*s*) against an average mutation affecting the TE number (i.e., transposition or excision) (Barton 1990; Crow 1993). Hence, if selection against TEs is present, it is very weak, with 2*N*_e_*s *=* *1.32. By simultaneously bootstrapping the MA and natural strains 10,000 times, we estimated that the 95% confidence interval of 2*N*_e_*s* is from 0.87 to 2.19.

In computing *V*_g_, we noticed a significant, negative correlation (*r *=* *−0.43, *P *=* *0.004) between the number of detected TEs from a natural strain of yeast and the number of scaffolds of its genome assembly (Bleykasten-Grosshans et al. 2013), suggesting that assembly quality might have affected the number of TE detected. However, using various cutoffs of the number of scaffolds (<10^4^, <10^3^, and <10^2^) to filter out strains with potentially unreliable TE estimates, we found *V*_g_/*V*_m_ to vary in the narrow range between 1.24 × 10^7^ and 1.69 × 10^7^. Thus, the above inferences based on the *V*_g_/*V*_m_ of 1.51 × 10^7^ estimated from all 41 natural strains are reliable. To investigate the robustness of the *V*_g_ estimate to the specific yeast strains considered, we estimated *V*_g_ based on all TEs previously identified from 38 natural *S. cerevisiae* strains (Carr et al. 2012). These 38 strains include most of the main lineages of *S. cerevisiae* (Maclean et al. 2017), but overlap with the 41 strains in Bleykasten-Grosshans et al.’s study by only five strains, so can be treated as an independent data set. We found *V*_g_/*V*_m_ to equal 1.39 × 10^7^ when these 38 strains were used to estimate *V*_g_. Thus, our inference of selection against TE is robust to different natural yeast strains considered.

## Discussion

Bast et al. (2019) reported that asexual experimental evolution of yeast caused a reduction in its TE number and explained the reduction by an adaptive increase in the TE excision rate under asexuality. In this work, we showed that some of the genomic data analyzed by Bast et al. (2019) had low sequencing coverages that rendered their TE number estimation unreliable and that there was no significant TE number reduction upon the removal of samples with low coverages. Analyses of data from two additional yeast experimental evolution studies confirmed that the TE number is stable in both sexual and asexual populations during experimental evolution. Results from population genetic simulations under more realistic parameters demonstrated that Bast et al.’s model cannot explain the observed yeast TEs. As such, the experimental evolution studies have not helped resolve the question of the net impact of sex on the genomic TE load. Given that yeast undergoes a sexual cycle per thousand asexual generations in nature, to investigate the impact of sexual reproduction on the genomic TE load in yeast, perhaps one would need to design experimental evolution where the frequency of sexual reproduction is much higher than once per 60 to 90 asexual generations that has been used.

Our estimation from yeast MA lines showed that the TE excision rate is only moderately lower than the transposition rate and that the two rates are not significantly different. Furthermore, the variance of the TE number among natural strains is only slightly below the neutral expectation and the estimated coefficient of selection against TEs is below 1/*N*_e_. Therefore, selective purging of deleterious TEs is very weak in yeast. Consequently, the hypothesized benefit of sex in enhancing the efficacy of selective purging of deleterious TEs may be essentially absent in yeast, which would push the net impact of sex toward promoting TE’s proliferation. Nevertheless, at least in *Drosophila*, the transposition rate is one to two orders of magnitude higher than the excision rate (Maside et al. 2000; Adrion et al. 2017). It is unclear whether the relatively high rate of TE excision in yeast is intrinsic or is a result of natural selection promoting excision in large, primarily asexual natural populations of yeast. Appropriately designed sexual and asexual long-term experimental evolution of yeast may help answer these fundamental questions about the impact of sex on the evolutionary dynamics of TEs.

## Materials and Methods

### Genomic Data from Yeast Experimental Evolution Studies

Data from three experimental evolution studies were analyzed in the present work. The first data set, previously analyzed by Bast et al. (2019), were generated from experimental evolution of a yeast strain derived from the W303 strain (McDonald et al. 2016). Briefly, McDonald et al. evolved four sexual and four asexual populations in yeast extract peptone dextrose (YPD) media for about 1,000 generations. Sexual populations underwent one sexual cycle per 90 asexual generations. Paired-end Illumina reads were generated for all lines at the haploid stage every 90 generations for a total of 11 sequenced time points per line.

The second data set was generated from the experimental evolution of the strain DBY15084 (a haploid strain derived from W303) in YPD for 1,000 generations (Lang et al. 2013). Paired-end Illumina reads were generated with a 100× genomic coverage on average. Each of the 40 populations was sequenced approximately every 80 generations for a total of 12 time points.

The third data set was generated from the experimental evolution of six sexual and six asexual populations (originating from diploids derived from W303) under a fluctuating environment for 1,440 generations (Leu et al. 2020). The sexual lineages underwent one sexual cycle per 60 asexual generations. Three sexual and three asexual diploid populations were sequenced to a 90× average coverage at approximately every 60 generations.

### Genomic Data from the Yeast MA Study

Liu and Zhang (2019) used the diploid BY4743 yeast strain to establish 168 MA lines in seven different solid media: yeast-extract–peptone–dextrose (YPD), yeast-extract–peptone–xylose (YPX), yeast-extract–peptone–lactose (YPL), yeast–nitrogen-base–dextrose (YNB), YPD with 6 mM CuSO4 (CuSO4), YPD with 100 mM LiCl (LiCl), and YPD with 1 M NaCl (NaCl), with 24 parallel lines per medium (one line in LiCl died out in MA). All MA lines were passaged by single-cell colony transfers, where a randomly selected average-size colony was streaked onto a new plate. They kept the total number of cell divisions in all MA lines to 1,000, corresponding to 60 bottlenecks in each medium. Paired-end Illumina reads were generated for the ancestor and 167 end colonies from the MA lines. The genome coverage was approximately 199× for the ancestor and ranged from 56× to 143× for the 167 MA lines. These data were used to estimate TE transposition and excision rates in the near absence of selection.

### Genomic TE Load

Illumina sequencing reads were mapped to the *S. cerevisiae* reference genome (version R64-2-1) or W303 reference genome by Burrows-Wheeler Aligner 0.7.17 with standard parameters (Li and Durbin 2009). The reference genome of the W303 strain was retrieved from Lang et al. (2013). Aligned reads were processed and sorted using SAMtools v1.8 (Li et al. 2009) and Picard v2.16.0 (http://broadinstitute.github.io/picard/, last accessed March 20, 2021). Per-base coverage was calculated using bedTools genomecov with parameters set to -ibam -d (Quinlan and Hall 2010). Following Bast et al. (2019), we defined the genomic sequencing coverage as the median coverage of all bases in the genome.

A curated and updated TE library that contained all consensus sequences of all TE families found in *S. cerevisiae* was downloaded from fig. S1 of Carr et al. (2012). With this library, we identified TEs in the corresponding reference genome using RepeatMasker v4.1.0 (http://www.repeatmasker.org, last accessed March 20, 2021) with parameters set to -nolow -lib -gccalc -s -cutoff 200 -no_is -nolow -norna -gff -u. We estimated the TE load by the weighted number of bases stemming from TE regions divided by the weighted number of bases of the genome, where the weight of a base was the number of hits it received from sequencing reads. This estimation is expected to be slightly more accurate than the fraction of reads mapped to TEs, which Bast et al. (2019) used to estimate the TE load, because a read could span the boundary between a TE region and its neighboring non-TE region. Because extrachromosomal DNA does not harbor TEs, we removed reads mapped to mitochondrial DNA and 2-micron plasmids before any analysis.

### Detection of Individual TEs Using RelocaTE2

We identified TEs in a genome using RelocaTE2, which detects reference and nonreference TEs using split-reads and discordant reads. Reference TEs refer to TEs that exist in the reference genome, whereas nonreference TEs do not exist in the reference genome. Compared with RelocaTE2, the tool McClintock (Nelson et al. 2017), which Bast et al. (2019) used, appeared to make more errors. The following factors may explain their different performances. First, the McClintock pipeline combines six TE detection programs. Although most of these programs use either split reads or paired reads to infer TEs, RelocaTE2 uses both split reads and discordant paired reads to infer TEs, improving the sensitivity and specificity. Second, because using paired reads to infer TE insertions is less accurate than using split reads, combining the results from six programs in McClintock is problematic. For example, TEMP (Zhuang et al. 2014), one of the six programs in McClintock, predicts TE insertion sites to a resolution of 393 ± 199 bases, making it difficult to differentiate among nearby insertion sites. By contrast, RelocaTE2 predicts TE insertion junctions to a much finer resolution (3 ± 1 bases). Third, some programs in McClintock have high false-negative rates (e.g., ngs_te_mapper), whereas some other programs have high false-positive rates (e.g., PoPoolationTE) (Nelson et al. 2017).

Briefly, the workflow of RelocaTE2 is initiated by using BLAT to align sequencing reads to the TE library. A read with similarity to TEs is called an informative read. Informative reads that partially match TE boundaries are trimmed to remove the TE regions and are then denoted as junction reads. Informative reads that completely match TEs are discarded, whereas their read pairs that do not match TEs are denoted as supporting reads. Junction reads, untrimmed versions of junction reads, and supporting reads are all aligned to the reference genome using BWA. The mapping positions of the junction reads are used to define TE transposition sites. Reference TEs are called based on the TE sequences in the reference genome identified by RepeatMasker, whereas the remaining TEs are nonreference TEs. Low-quality reference TEs are filtered out by removing TE transposition sites supported by <5 (or two for McDonald et al.’s data) junction and supporting reads in total. Low-quality nonreference TEs are filtered out by removing transposition sites supported by <5 (or two for McDonald et al.’s data) junction reads in total and by removing TE transposition sites within ten bases from a reference TE.

We evaluated the impact of genomic sequencing coverage on TE detection in the following way. The reads in the sample with the highest sequencing coverage were downsampled to the levels of coverage of the other samples, followed by TE detection by RelocaTE2. We then performed a regression of the number of detected TEs on the sequencing coverage using the shape constrained additive model (SCAM) (Pya and Wood 2015) by the R package SCAM (Pya 2020). SCAM is an extension of the generalized additive model (GAM) that enables the imposition of a monotonic increase or decrease on the P-splines to incorporate constraints on the shape of the GAM. Based on the regression line, we computed a correction factor for each coverage level by dividing the number of detected TEs in the original sample before downsampling to the number of detected TEs on the regression line for the coverage concerned. The number of TEs for an actual sample is the observed TE number from that sample multiplied by the correction factor for the corresponding coverage.

### Detection of TE Excisions in Diploid Genomes

To detect TEs that are present in the reference genome but are heterozygously or homozygously deleted in a diploid sample genome, we identified read pairs for which the distance between the mapped locations of the two reads was between 1 and 20 kb (supplementary fig. S6*c*, Supplementary Material online). We then clustered read pairs supporting the same event. Finally, we examined whether the deleted genomic region spans one or more known TEs annotated in the reference genome. A series of cleaning steps were used to filter out low-quality candidate excisions. Briefly, we removed reads with mapping quality (MAPQ) lower than 20 (i.e., the probability of correct mapping <0.99) and removed excisions supported by <4 read pairs. We confirmed the excisions by loading the mapped reads on IGV.

### Genome-Wide Rates of Transposition and Excision

The genome-wide rate of TE transposition or excision per full-length TE per generation is the number of transposition or excision events divided by (50 × 2 × 167 × 1,000), where 50 × 2 is the total number of full-length TEs per diploid genome, 167 is the number of MA lines, and 1,000 is the number of generations of MA.

### Effective Population Size of *S. cerevisiae*

Yeast point mutation rate has been estimated from MA lines to be *μ* = 1.95 × 10^−10^ per site per generation in YPD (Liu and Zhang 2019). In a species-wide population genomic survey of *S. cerevisiae* (Maclean et al. 2017), it was found that the nucleotide diversity per site (*π*) is substantially lower at nonsynonymous (0.0014), intronic (0.0027), and intergenic (0.0037) sites than at synonymous sites (0.0091). We thus assume that synonymous polymorphisms are largely neutral. The effective population size is then estimated by *N*_e_* *=* π*/(4 *μ*)* *=* *1.17 × 10^7^.

### Population Genetic Simulations

We used the simulation program TEAscus in Bast et al.’s study to trace the evolution of TEs in asexual and sexual populations. The simulation was performed for haploid individuals in an asexual population; sexual cycles were added by fusion of two haploid individuals followed by meiosis and production of haploid individuals in sexual populations. Unless otherwise noted, our simulation parameters were identical to those in Bast et al. (2019). That is, population size* *=* *10^5^, starting number of TEs in the genome* *=* *50, number of chromosomes* *=* *16, genome size* *=* *8,000 loci (i.e., 500 loci per chromosome), recombination rate* *=* *5.6 crossovers per chromosome per meiosis, transposition rate* *=* *10^−5^ per TE per generation, excision rate* *=* *10^−5^ per TE per generation, and selection coefficient against TE* *=* *5 × 10^−4^ per TE per generation. We performed ten replicates of each simulation, using the mean TE number in the population measured per 90 generations as the output.

## Supplementary Material

Supplementary data are available at *Molecular Biology and Evolution* online.

## Supplementary Material

msab073_Supplementary_Data
