# Supplementary material for: Asexual Experimental Evolution of Yeast Does Not Curtail Transposable Elements
Source: Mol Biol Evol. 2021 Mar 15;38(7):2831–42. doi: 10.1093/molbev/msab073 (PMC8233515; doi:10.1093/molbev/msab073)
Supplement: msab073_Supplementary_Data [file msab073_supplementary_data.pdf]

Supplementary materials for

"**Asexual experimental evolution of yeast does not curtail transposable elements**"

P. Chen & J. Zhang (jianzhi@umich.edu)

The supplementary materials include:

Tables S1-S3

Legends of supplementary figures

Figures S1-S6

Table S1. Yeast genomic sequencing data used in this study.

Reproductive mode	BioProject ID	Reference
Asexual + Sexual	PRJNA308843	McDonald *et al.* (2016)
Asexual	PRJNA205542	Lang *et al.* (2013)
Asexual + Sexual	PRJNA530331	Leu *et al.* (2020)
Asexual	PRJNA510430	Liu and Zhang (2019)

Table S2. Genomic sequencing coverage and TE number of each population in McDonald *et al.*'s data.

Strain	Generation	Mating	Coverage	TE #	TE # after correction
2A5A	0	Sexual	52	443	449.78
2A5A	180	Sexual	62	447	452.68
2A5A	270	Sexual	75	446	450.71
2A5A	360	Sexual	38	443	453.05
2A5A	450	Sexual	44	442	450.22
2A5A	540	Sexual	34	443	454.84
2A5A	630	Sexual	53	442	448.63
2A5A	720	Sexual	71	445	449.95
2A5A	810	Sexual	25	431	450.52
2A5A	90	Sexual	21	438	466.03
2A5A	900	Sexual	54	444	450.52
2A5A	990	Sexual	42	447	455.83
2C5C	0	Sexual	38	434	443.85
2C5C	180	Sexual	6	327	550.14
2C5C	270	Sexual	64	448	453.51
2C5C	360	Sexual	271	453	453.22
2C5C	450	Sexual	79	451	455.53
2C5C	540	Sexual	160	452	454.05
2C5C	630	Sexual	31	387	398.90
2C5C	720	Sexual	106	450	453.40
2C5C	810	Sexual	120	451	453.98
2C5C	90	Sexual	92	448	451.90
2C5C	900	Sexual	119	453	456.02
2C5C	990	Sexual	48	444	451.44
2D5D	0	Sexual	94	450	453.83
2D5D	180	Sexual	78	447	451.55
2D5D	270	Sexual	160	449	451.04
2D5D	360	Sexual	89	450	454.04
2D5D	450	Sexual	46	448	455.89
2D5D	540	Sexual	208	454	455.20
2D5D	630	Sexual	104	443	446.41
2D5D	720	Sexual	74	445	449.76
2D5D	810	Sexual	41	443	452.04
2D5D	90	Sexual	109	453	456.33
2D5D	900	Sexual	19	424	456.82
2D5D	990	Sexual	40	440	449.28
2F5F	180	Sexual	256	447	447.44

2F5F	270	Sexual	245	452	452.62
2F5F	360	Sexual	93	449	452.86
2F5F	450	Sexual	253	454	454.49
2F5F	540	Sexual	144	447	449.36
2F5F	630	Sexual	285	447	447.00
2F5F	720	Sexual	268	453	453.26
2F5F	810	Sexual	73	443	447.80
2F5F	90	Sexual	188	444	445.50
2F5F	900	Sexual	67	447	452.26
2F5F	990	Sexual	69	447	452.11
3C	180	Asexual	116	450	453.09
3C	270	Asexual	128	448	450.75
3C	360	Asexual	145	451	453.36
3C	450	Asexual	45	449	457.12
3C	540	Asexual	62	447	452.68
3C	630	Asexual	97	447	450.69
3C	720	Asexual	126	452	454.83
3C	810	Asexual	108	454	457.36
3C	90	Asexual	36	446	456.95
3C	900	Asexual	12	170	201.50
3C	990	Asexual	11	78	95.33
3D	0	Asexual	10	397	504.30
3D	180	Asexual	57	437	443.05
3D	270	Asexual	30	366	377.86
3D	360	Asexual	63	448	453.60
3D	450	Asexual	47	448	455.70
3D	540	Asexual	66	449	454.36
3D	630	Asexual	36	419	429.28
3D	720	Asexual	34	420	431.22
3D	810	Asexual	40	426	434.99
3D	90	Asexual	0	2	44.89
3D	900	Asexual	37	433	443.21
3D	990	Asexual	34	432	443.54
6C	180	Asexual	61	450	455.81
6C	270	Asexual	81	449	453.41
6C	360	Asexual	75	449	453.74
6C	450	Asexual	86	450	454.17
6C	540	Asexual	34	446	457.92
6C	720	Asexual	10	118	149.89
6C	810	Asexual	5	107	203.66
6C	90	Asexual	48	449	456.53

6C	900	Asexual	19	283	304.91
6C	990	Asexual	11	232	283.55
6D	180	Asexual	38	446	456.12
6D	270	Asexual	32	445	458.02
6D	360	Asexual	37	432	442.19
6D	450	Asexual	29	445	460.27
6D	540	Asexual	105	451	454.44
6D	630	Asexual	40	434	443.16
6D	720	Asexual	11	344	420.44
6D	810	Asexual	15	368	411.47
6D	90	Asexual	36	447	457.97
6D	900	Asexual	29	425	439.59
6D	990	Asexual	15	390	436.07

Table S3. TE transposition (insertion) and excision (deletion) events identified from the MA lines of Liu and Zhang's data.

Environment	Population ID	Mutation	Chromosome	Position	Ty element
$CuSO_4$	10	Deletion	chrVII	535000-542000	TY1
$CuSO_4$	17	Insertion	chrXI	302813-302815	TY2
LiCl	1	Deletion	chrIV	877000-885000	TY1
LiCl	2	Deletion	chrXV	594000-601000	TY1
LiCl	9	Deletion	chrXVI	56000-63000	TY1
LiCl	15	Deletion	chrXIII	372000-379000	TY1
LiCl	18	Deletion	chrXVI	844000-851000	TY1
LiCl	22	Deletion	chrIV	1095000-1102000	TY1
NaCl	1	Deletion	chrIV	878000-885000	TY1
NaCl	2	Deletion	chrI	160000-167000	TY1
NaCl	7	Insertion	chrXV	113707-113709	TY2
NaCl	9	Insertion	chrI	180990-180992	TY2
NaCl	11	Insertion	chrIX	210314-210318	TY1
NaCl	15	Insertion	chrVI	205082-205086	TY1
NaCl	15	Insertion	chrXV	980507-980511	TY1
NaCl	18	Deletion	chrXVI	844000-851000	TY1
NaCl	18	Deletion	chrXVI	850000-857000	TY1
NaCl	20	Insertion	chrXIV	560988-560992	TY1
NaCl	21	Deletion	chrXV	703000-710000	TY2
NaCl	24	Insertion	chrXVI	289172-289176	TY1
YNB	5	Insertion	chrVIII	238127-238129	TY2
YNB	6	Insertion	chrVII	736188-736190	TY2
YNB	21	Insertion	chrXVI	769403-769407	TY2
YPD	11	Insertion	chrXV	274526-274530	TY2
YPD	18	Insertion	chrXIV	104331-104335	TY1
YPL	1	Insertion	chrVII	531411-531413	TY2
YPL	3	Insertion	chrXIII	808575-808577	TY2
YPL	7	Insertion	chrVII	701176-701180	TY1
YPL	11	Insertion	chrVI	101035-101037	TY2
YPL	11	Insertion	chrXI	308602-308604	TY2
YPL	14	Deletion	chrIV	1206000-1213000	TY1
YPL	19	Insertion	chrIV	1095681-1095685	TY1
YPL	24	Deletion	chrXIII	372000-379000	TY1
YPX	4	Insertion	chrX	354139-354141	TY2
YPX	8	Insertion	chrI	184661-184663	TY2
YPX	21	Deletion	chrVIII	543000-550000	TY1

Legends of supplementary figures

Fig. S1. The genomic TE load at each time point for individual asexual lines in McDonald *et al.*'s experimental evolution study. Solid and open dots show samples with genome sequencing coverage >20× and < 20×, respectively. The blue dashed line shows the linear regression. Pearson's correlation (*r*) between TE load and number of generations of experimental evolution is shown along with the *P* value.

Fig. S2. Estimated numbers of TEs of individual asexual lines over McDonald *et al.*'s experimental evolution. (a-c) Numbers of full-length TEs (a), reference TEs (b), and non-reference TEs (c) detected from individual subsamples created by downsampling the reads from sexual population 2F5F at 630 generations to the coverages of all real samples. The red dashed line is the SCAM regression. The vertical line indicates the coverage of 20. (d-e) Estimated numbers of TEs (d) and full-length TEs (e) of individual samples upon the regression-based correction for low coverage. Solid and open dots show samples with genomic sequencing coverage >20× and <20×, respectively. The blue dashed line shows the linear regression. Pearson's correlation (*r*) between TE number and number of generations of experimental evolution is shown along with the *P* value.

Fig. S3. Relationships between the genomic sequencing coverage and various measures of data quality. (a) Mean fragment size of each sequencing library. (b) Fraction of mapped reads (with mapping quality MAPQ higher than 10). (c) Mean Phred quality score of the last 50 bases in read1 and read2. (d) Relationship between the mean fragment size and corrected TE number. Each dot represents a population, and the dots are marked in four colors according to the genomic sequencing coverage and the TE count after the sequencing depth-based correction (20% of populations have corrected TE numbers below 443). Spearman's rank correlation (ρ) and *P*-value are shown.

Fig. S4. Sequencing coverages and TE numbers in Lang *et al.*'s 40 asexual experimental evolutionary lines. (a) Sequencing coverages of the 40 lines over time. In each box plot, the lower and upper edges of a box represent the first (qu_1) and third (qu_3) quartiles, respectively, the horizontal line inside the box indicates the median (md), the whiskers extend to the most extreme

values inside inner fences, md ± 1.5($qu_3 - qu_1$), and the circles represent values outside the inner fences (outliers). No significant correlation is found between coverage and number of generations of evolution ($\rho = -0.04$, $P = 0.39$). (b-e) Numbers of TEs (b), reference TEs (c), non-reference TEs (d), and full-length TEs (e) detected from individual subsamples created by downsampling the reads from population BYS1_E03 at 1000 generations to the coverages of all real samples. The red dashed line is the SCAM regression. Spearman's correlation (ρ) between TE number and sequencing coverage is shown along with the *P* value.

Fig. S5. Genomic TE load and various TE numbers in Leu *et al.*'s sexual and asexual experimental evolution lines. (a) The genomic TE load of individual lines over time. Solid and open dots show samples with genomic sequencing coverage >20× and <20×, respectively. Pearson's correlation (*r*) between TE load and number of generations of experimental evolution is shown along with the *P* value. the dashed line shows the linear regression. (b) Genomic sequencing coverage of sexual and asexual populations. In each box plot, the lower and upper edges of a box represent the first (qu_1) and third (qu_3) quartiles, respectively, the horizontal line inside the box indicates the median (md), the whiskers extend to the most extreme values inside inner fences, md ± 1.5($qu_3 - qu_1$), and the circles represent values outside the inner fences (outliers). The *P*-value is based on Wilcoxon rank-sum test. (c-f) Numbers of TEs (c), full-length TEs (d), reference TEs (e), and non-reference TEs (f) detected from individual subsamples created by downsampling the reads from population sexual_1 at 1,440 generations to the coverages of all real samples. The red dashed line is the SCAM regression. (g) Estimated numbers of TEs of individual samples upon the regression-based correction for low coverage. Symbols follow panel a.

Fig. S6. Detecting TE transpositions and excisions from diploid MA lines. (a) Integrated Genomics Viewer (IGV) display of a TE transposition event in the MA line 17 in the $CuSO_4$ environment at the region chrXI:302813-302815. The ancestor and sample $CuSO_4$-17 are presented in two separate groups. The upper part of each panel is a histogram representing the read depth at each location, while the lower part is a graphical view of some reads aligned to that location. A colored read has its paired read aligned to a different chromosome, and the color indicates that chromosome. (b) IGV display of the deletion at the region chrXII:732282-734685

in multiple MA lines that is not a genuine excision event. Reads that are colored red have larger-than-expected inferred insert size, so indicate a deletion. The ancestor, LiCl-4, and LiCl-5 are represented in three separate groups. (c) Diagram depicting the detection of TE excision based on paired reads. Excision is detected when the two reads of a read pair are respectively mapped to the two flanking regions of a TE in the reference genome.

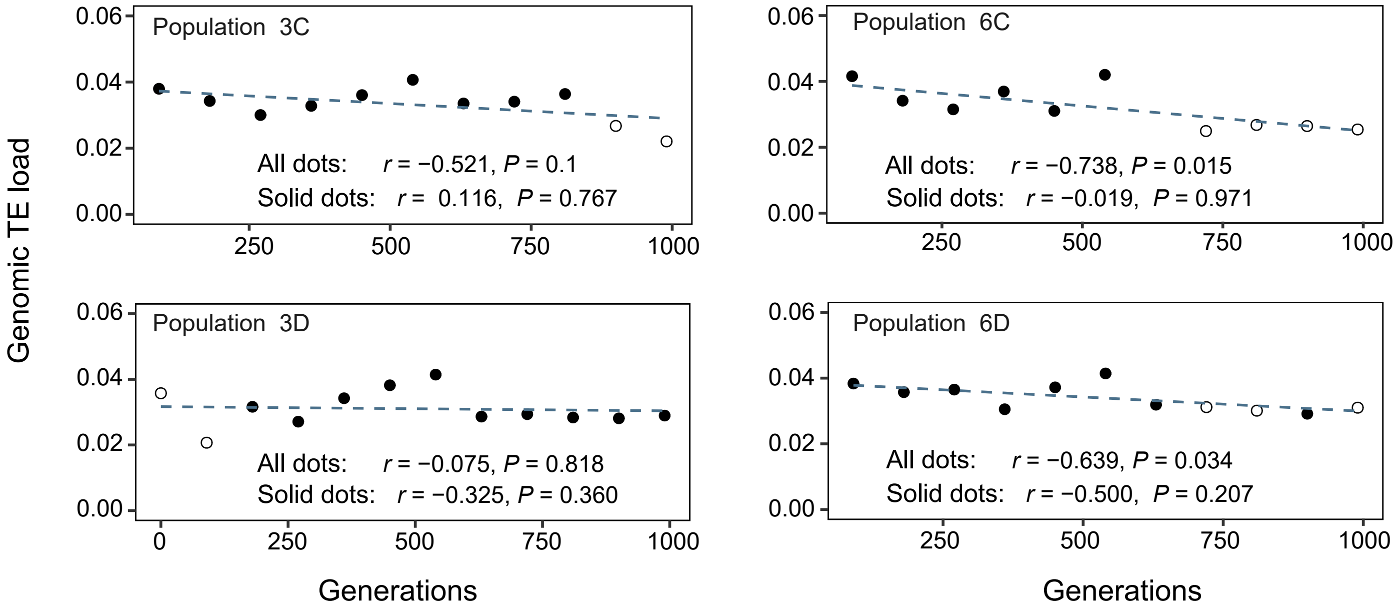
Genomic TE load
Population 3C
All dots: r = −0.521, P = 0.1
Solid dots: r = 0.116, P = 0.767
Population 6C
All dots: r = −0.738, P = 0.015
Solid dots: r = −0.019, P = 0.971
Population 3D
All dots: r = −0.075, P = 0.818
Solid dots: r = −0.325, P = 0.360
Population 6D
All dots: r = −0.639, P = 0.034
Solid dots: r = −0.500, P = 0.207
Generations
Generations

Fig. S1

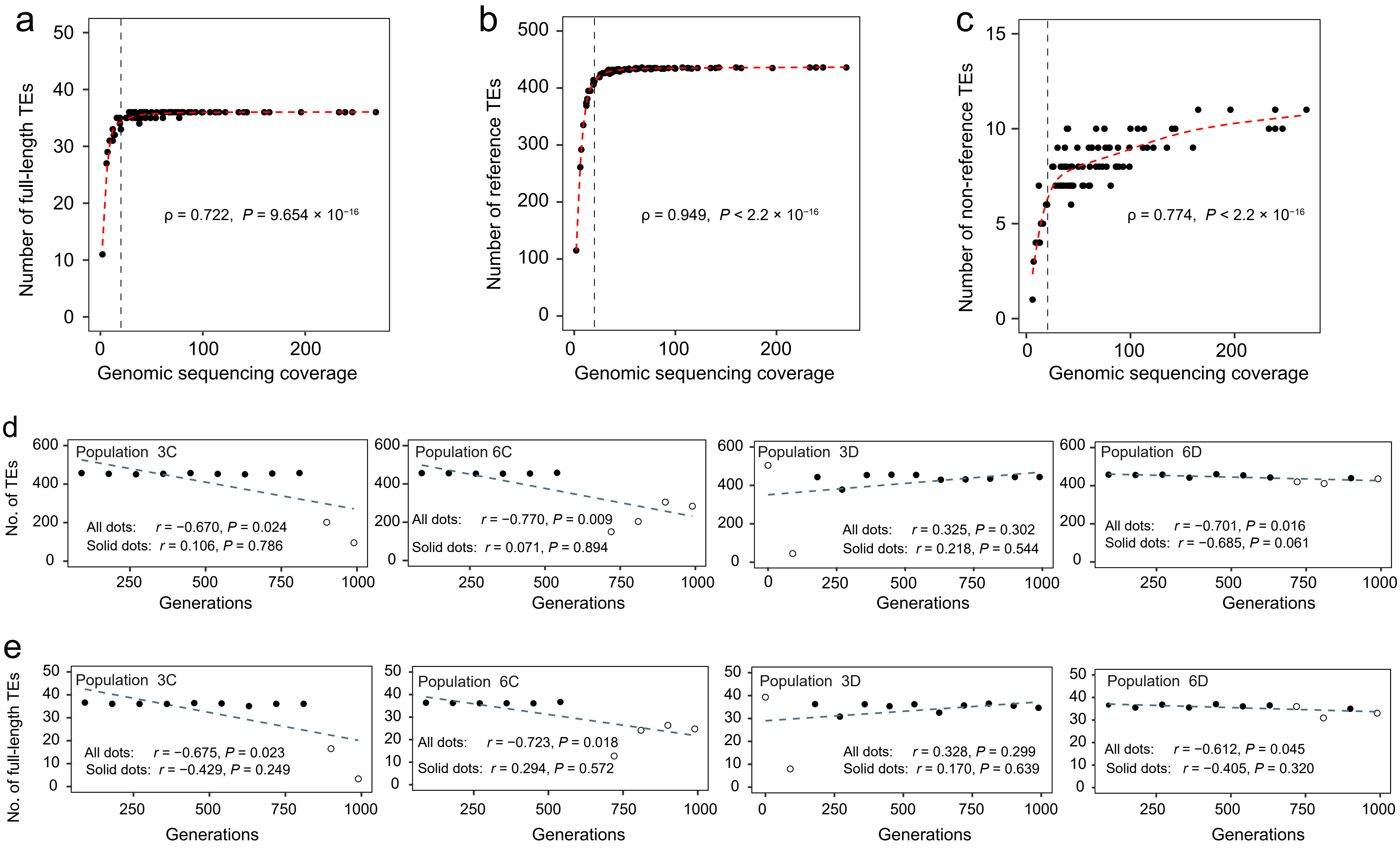

a
Number of full-length TEs
Genomic sequencing coverage
ρ = 0.722, P = 9.654 × 10−16
b
Number of reference TEs
Genomic sequencing coverage
ρ = 0.949, P < 2.2 × 10−16
c
Number of non-reference TEs
Genomic sequencing coverage
ρ = 0.774, P < 2.2 × 10−16
d
No. of TEs
Population 3C
All dots: r = −0.670, P = 0.024
Solid dots: r = 0.106, P = 0.786
Population 6C
All dots: r = −0.770, P = 0.009
Solid dots: r = 0.071, P = 0.894
Population 3D
All dots: r = 0.325, P = 0.302
Solid dots: r = 0.218, P = 0.544
Population 6D
All dots: r = −0.701, P = 0.016
Solid dots: r = −0.685, P = 0.061
Generations
e
No. of full-length TEs
Population 3C
All dots: r = −0.675, P = 0.023
Solid dots: r = −0.429, P = 0.249
Population 6C
All dots: r = −0.723, P = 0.018
Solid dots: r = 0.294, P = 0.572
Population 3D
All dots: r = 0.328, P = 0.299
Solid dots: r = 0.170, P = 0.639
Population 6D
All dots: r = −0.612, P = 0.045
Solid dots: r = −0.405, P = 0.320
Generations

Fig. S2

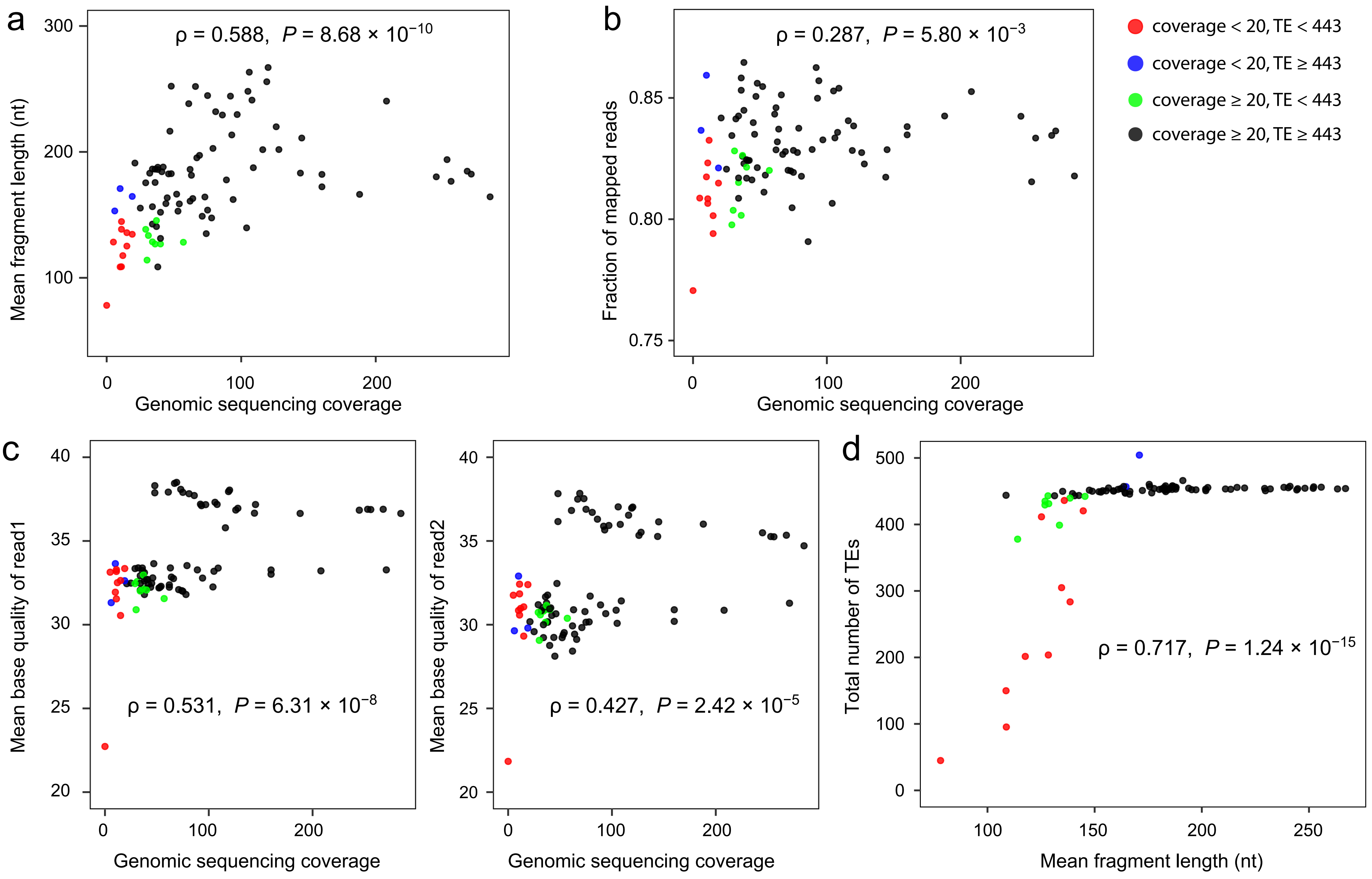

a
ρ = 0.588, P = 8.68 × 10^−10
Mean fragment length (nt)
Genomic sequencing coverage
b
ρ = 0.287, P = 5.80 × 10^−3
Fraction of mapped reads
Genomic sequencing coverage
coverage < 20, TE < 443
coverage < 20, TE ≥ 443
coverage ≥ 20, TE < 443
coverage ≥ 20, TE ≥ 443
c
Mean base quality of read1
ρ = 0.531, P = 6.31 × 10^−8
Genomic sequencing coverage
Mean base quality of read2
ρ = 0.427, P = 2.42 × 10^−5
Genomic sequencing coverage
d
Total number of TEs
ρ = 0.717, P = 1.24 × 10^−15
Mean fragment length (nt)

Fig. S3

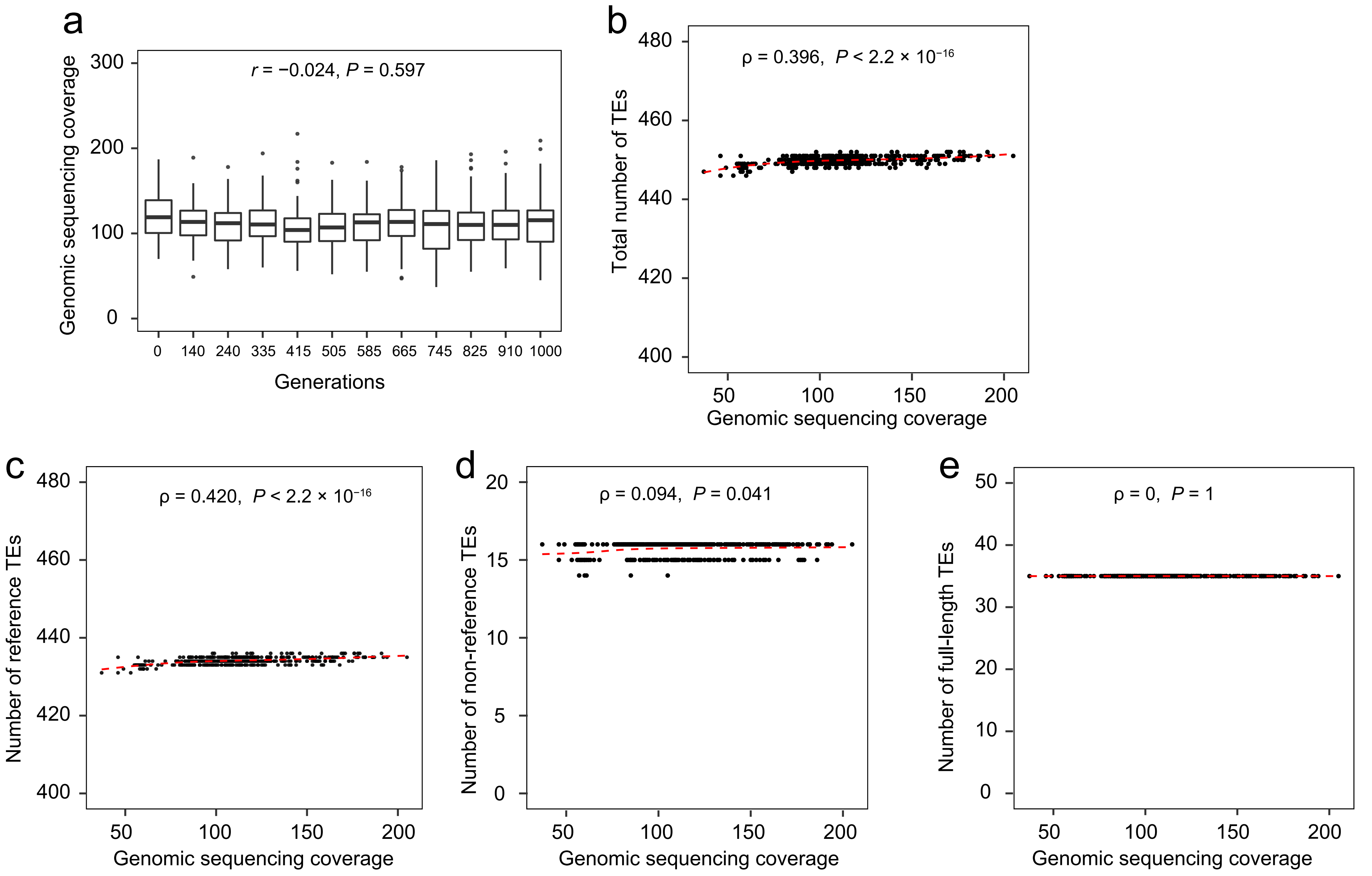
a
r = −0.024, P = 0.597
Genomic sequencing coverage
300
200
100
0
0 140 240 335 415 505 585 665 745 825 910 1000
Generations
b
ρ = 0.396, P < 2.2 × 10⁻¹⁶
Total number of TEs
480
460
440
420
400
50 100 150 200
Genomic sequencing coverage
c
ρ = 0.420, P < 2.2 × 10⁻¹⁶
Number of reference TEs
d
ρ = 0.094, P = 0.041
Number of non-reference TEs
e
ρ = 0, P = 1
Number of full-length TEs

Fig. S4

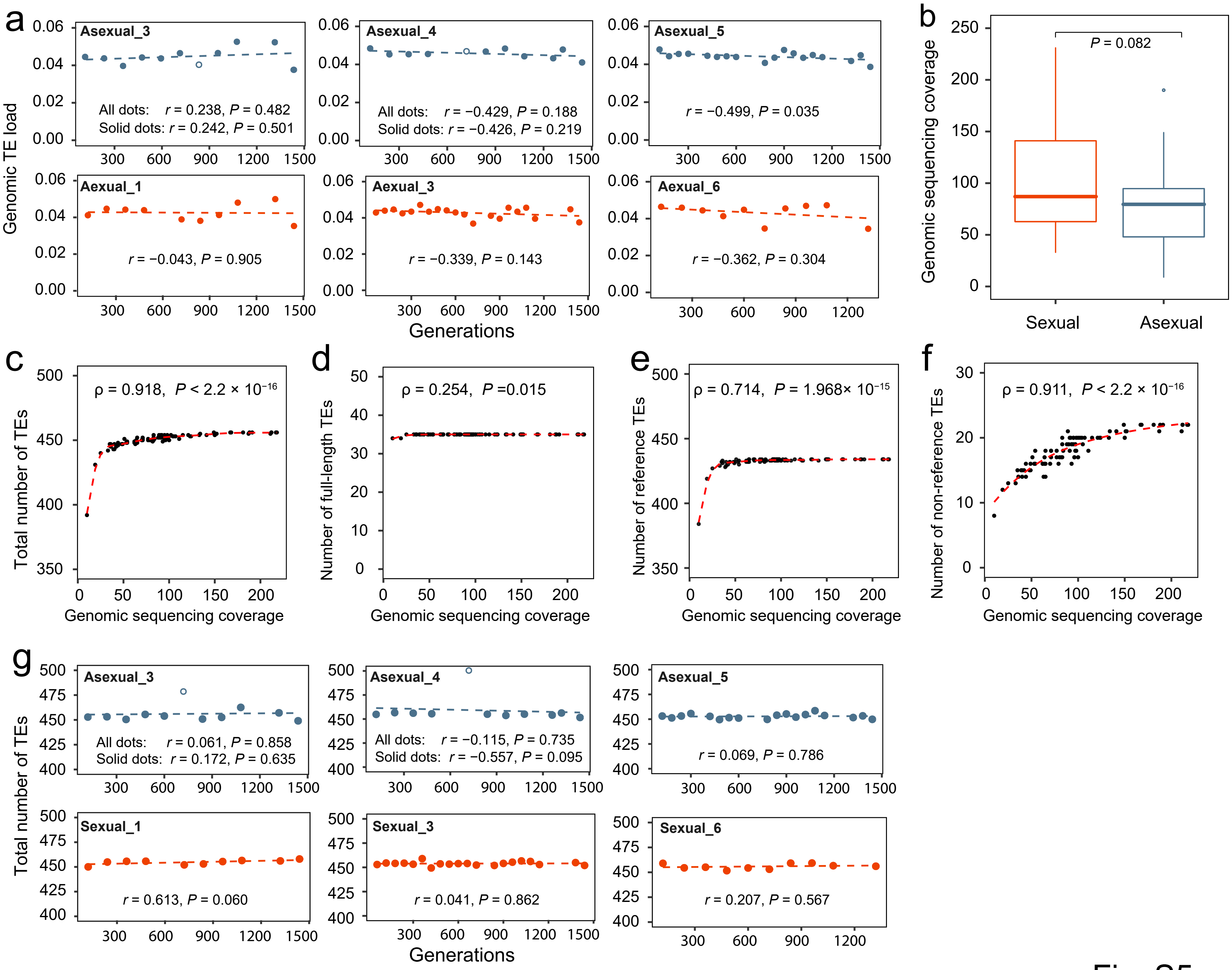
a
Asexual_3
All dots: r = 0.238, P = 0.482
Solid dots: r = 0.242, P = 0.501
Asexual_4
All dots: r = −0.429, P = 0.188
Solid dots: r = −0.426, P = 0.219
Asexual_5
r = −0.499, P = 0.035
Genomic TE load
Aexual_1
r = −0.043, P = 0.905
Aexual_3
r = −0.339, P = 0.143
Aexual_6
r = −0.362, P = 0.304
Generations
b
P = 0.082
Genomic sequencing coverage
Sexual
Asexual
c
ρ = 0.918, P < 2.2 × 10−16
Total number of TEs
Genomic sequencing coverage
d
ρ = 0.254, P =0.015
Number of full-length TEs
Genomic sequencing coverage
e
ρ = 0.714, P = 1.968× 10−15
Number of reference TEs
Genomic sequencing coverage
f
ρ = 0.911, P < 2.2 × 10−16
Number of non-reference TEs
Genomic sequencing coverage
g
Asexual_3
All dots: r = 0.061, P = 0.858
Solid dots: r = 0.172, P = 0.635
Asexual_4
All dots: r = −0.115, P = 0.735
Solid dots: r = −0.557, P = 0.095
Asexual_5
r = 0.069, P = 0.786
Total number of TEs
Sexual_1
r = 0.613, P = 0.060
Sexual_3
r = 0.041, P = 0.862
Sexual_6
r = 0.207, P = 0.567
Generations

Fig. S5

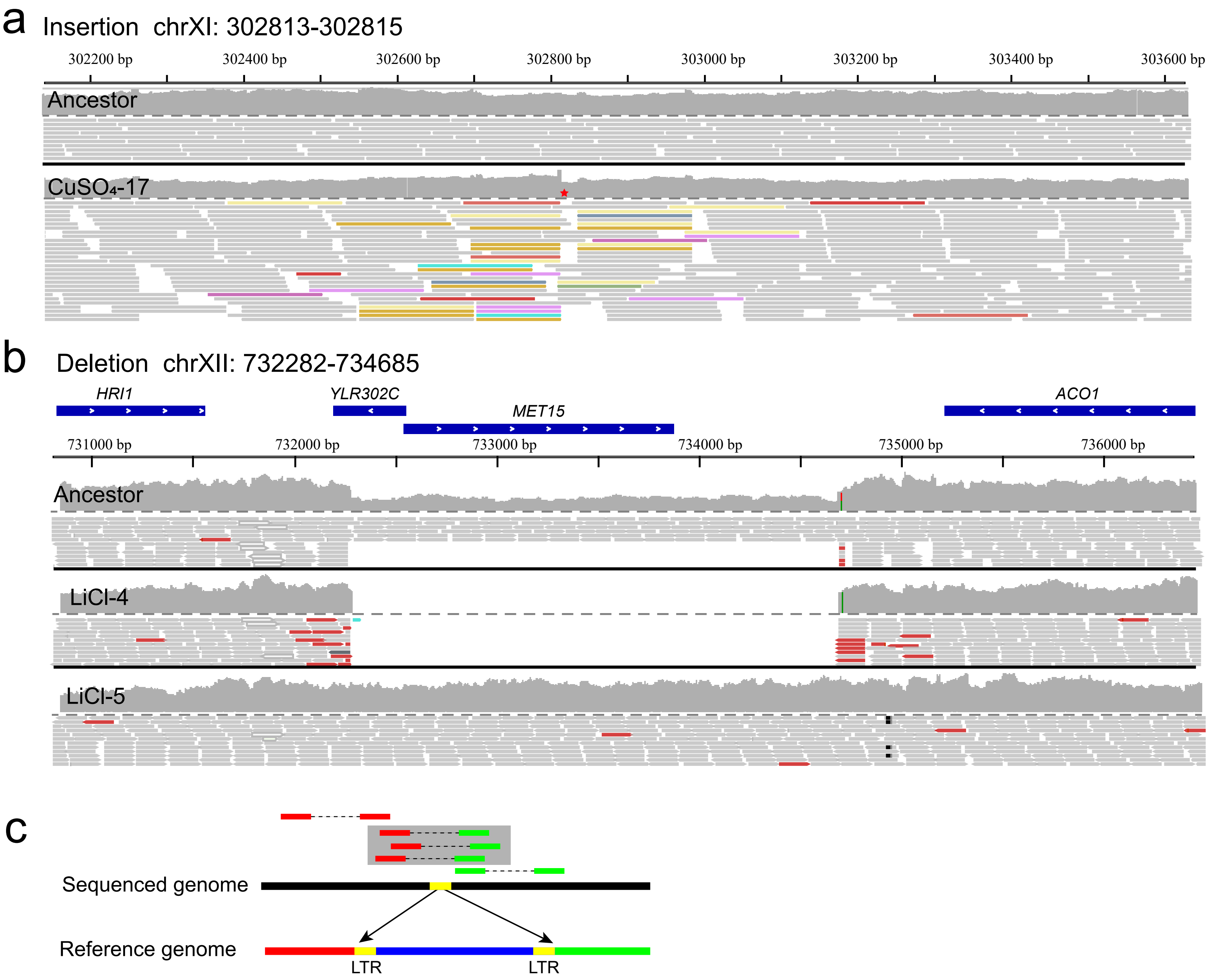
a Insertion chrXI: 302813-302815
302200 bp
302400 bp
302600 bp
302800 bp
303000 bp
303200 bp
303400 bp
303600 bp
Ancestor
CuSO4-17
b Deletion chrXII: 732282-734685
HRI1
YLR302C
MET15
ACO1
731000 bp
732000 bp
733000 bp
734000 bp
735000 bp
736000 bp
Ancestor
LiCl-4
LiCl-5
c
Sequenced genome
Reference genome
LTR
LTR

Fig. S6
